# Intra Uterine Amputation

**Published:** 1856-03

**Authors:** 


*Intra Uterine Amputation.*—At a late meeting of the surgical society of Paris, M. Huguier exhibited a child three years old, who was born without the hand and half the forearm on the right side. The limb looks as if it had been amputated about the middle, and the radius and ulna are easily felt.

